# Can bipolar transurethral enucleation of the prostate be a better alternative to the bipolar transurethral resection of the prostate? A prospective comparative study

**DOI:** 10.1097/MD.0000000000025745

**Published:** 2021-05-21

**Authors:** Mohammed Abdulwahab Al-radhi, Lo Ka Lun, Mohammed Safi, Abdullah Al-danakh, Khaled M Al-Kohlany, Amr Al-Najar, Hesham Al-sharani, Mahmoud Al-Azab, XianCheng Li, Chao Wang

**Affiliations:** a Department of Urology, Second Affiliated Hospital of Dalian Medical University, Liaoning Province, China; b The Chinese University of Hong Kong, Hong Kong; c Department of Oncology; d Department of Urology, First Affiliated Hospital of Dalian Medical University, Liaoning Province, China; e Medical College, Sana, a University Sana, a Yemen; f Saba University Sana, a Yemen; g Department of Immunology, Guangzhou Institute of Pediatrics, Guangzhou Women and Children's Medical Center, Guangzhou Medical University; h The First Affiliated Hospital of Jinzhou Medical University, Liaoning Province; i Soochow University, Soochow China.

**Keywords:** benign prostatic hyperplasia, bipolar transurethral enucleation of the prostate, bipolar transurethral resection of the prostate

## Abstract

To analyze the efficacy and safety between bipolar transurethral enucleation of the prostate (BipoLEP) and bipolar transurethral resection of the prostate (B-TURP).

One hundred twenty eight patients with benign prostatic hyperplasia were recruited and divided into group 1 (BipoLEP group, n = 72) and group 2 (B-TURP group, n = 56). The study period was from October 2016 to February 2019. All data parameters were prospectively collected and analyzed.

In these 2 groups, there were no significant differences of the mean ages (71.88 ± 6.54 years vs 73.05 ± 7.05 years, *P* = .407), prostate volumes (99.14 ± 9.5 mL vs 95.08 ± 10.93 mL, *P* = .302) and the mean operation times (93.7 ± 27.5 minutes vs 89.8 ± 22.4 minutes, *P* = .065). In BipoLEP group, it had more prostate tissue resected (64.2 ± 22.1 g vs 52.7 ± 28.6 g, *P* = .018), less duration of continuous bladder irrigation (20.7 ± 6.5 hours vs 29.6 ± 8.3 hours, *P* = .044), shorter catheterization time (4.3 ± 1.5 days vs 5.6 ± 2.1 days, *P* = .032), shorter hospitalization stay (5.2 ± 1.4 days vs 6.5 ± 1.9 days, *P* = .031) and less complications (3 cases vs 9 cases, *P* = .021). There were significant improvements in 3-month postoperative parameters, including: post void residual urine, maximum flow rate, International Prostatic Symptoms Scale, and quality of life in each group (p < 0.01). However, there were no significant differences of preoperative and 3-month postoperative parameters, including: post void residual urine, maximum flow rate, International Prostatic Symptoms Scale, and quality of life between these 2 groups (*P* > .05).

BipoLEP can produce a more radical prostatic resection with better safety profile and faster postoperative recovery. It may become a more favorable surgical alternative to the B-TURP, especially for the prostate larger than 80 g.

## 1 Introduction

Benign prostatic hyperplasia (BPH) is an age-dependent pathoanatomic condition with an initial histopathologic development after 40 years of age, and prevalence rates of approximately 50% and 90% by 60 and 85 years of age, respectively.^[1]^ Cell proliferation associated with BPH is comprised of both epithelial and stromal elements. BPH can cause bladder outlet obstruction, resulting in the lower urinary tract symptoms that are commonly classified as “obstructive symptoms” and “storage symptoms.” TURP is still the gold standard for surgical treatment of BPH but it is associated with more complications including bleeding and transurethral resection syndrome (TUR syndrome), particularly in the large prostate. So, open prostatectomy is still one of the treatment of choices for the prostate larger than 80 g.^[2,3]^

In recent years, with the rapid development of technology and equipment, there is increasing utilization of B-TURP in treating BPH. It uses the plasma kinetic system which has good coagulation function. As it uses normal saline as the irrigation fluid, it decreases the incidence of TUR syndrome. Previous studies also showed a promising result of B-TURP in treating bladder outlet obstruction that resulted from BPH.^[4–6]^

Bipolar transurethral enucleation of the prostate (BipoLEP) also showed its efficacy on variable prostate sizes ranged from 20 g to 250 g. ^[4,7]^ It has been well recognized and developed for many years with satisfactory result and low morbidity. So, in this study, we aimed to compare the efficacy and safety between BipoLEP and B-TURP in treating BPH.

## 2 Materials and methods

One hundred twenty eight patients from Department of Urology, The First Affiliated Hospital of Jinzhou Medical University, Jinzhou, China complaining of symptoms of BPH were recruited into the BipoLEP group (n = 72) and the B-TURP group (n = 56) in the period between October 2016 and November 2018. The study was approved by the ethics committee of The First Affiliated Hospital of Jinzhou medical University and the informed consent of patient was waived by the ethics committee due to the retrospective nature of the study design. All the operations were done by experienced urologists.

Methods surgical treatments of BPH include endoscopic surgery, open prostatectomy, transurethral surgery, laparoscopic/robotic surgery, and other novel treatments.

Endoscopic surgery: TURP is still the gold standard for treating BPH. The application of bipolar plasma electrocuting technology has improved the outcome of TURP. The procedure is mostly performed under the spinal anesthesia, the patient is in Lloyd Davis position during the procedure.

**Figure 1:**
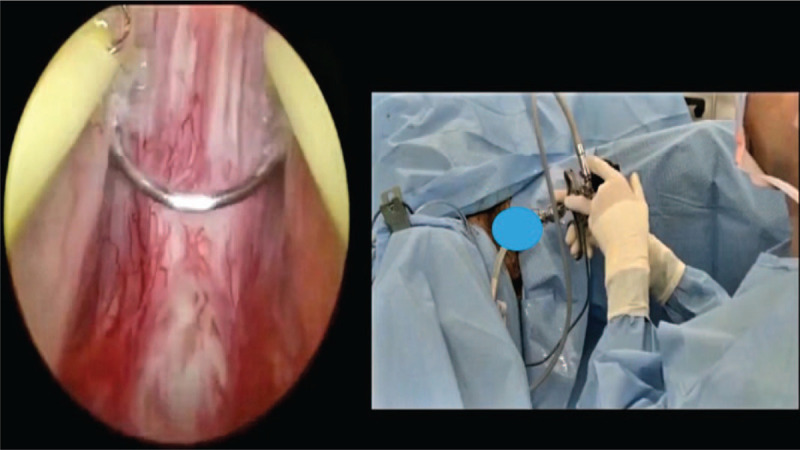
The verumontanum (landmark) should be observe in the beginning of the operation and the first incision must be done above and in the both sides the verumontanum in the shape of inverted v to avoid damaging the urethral sphincter.

**Figure 2:**
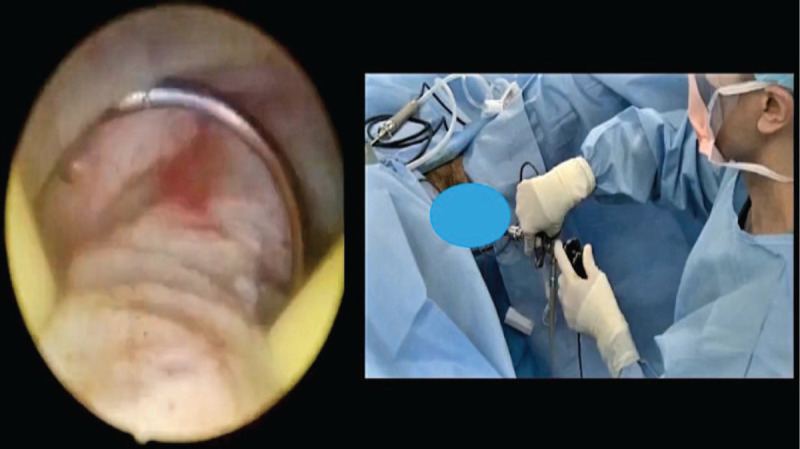
The space that formed between the prostate enucleated adenoma and the surgical capsule also the end point vessels that is suitable for coagulation.

In this study, we used Gyrus plasma bipolar endoscopic prostatectomy system in both groups. The power setting was 200/100 W. Warm normal saline was used as isotonic irrigation fluid. The irrigation pressure was 45 to 60 cmH2O.^[6]^ The procedure started by identifying the bilateral ureteric orifices, the bladder neck and the trigone. For BipoLEP, the first incision was made proximal to the verumontanum as shown in Figure 1, so as to avoid damaging the urethral sphincter. The incision should be cut deep enough down to the level of the surgical capsule (Fig. 2). The prostatic adenoma was enucleated in the retrograde fashion towards the bladder neck by the tip of resectoscope. Apart from hemostasis, the resection loop was used to cut off the mucosa and adhesive fibers between the surgical capsule and the prostatic adenoma during the enucleation.

The enucleation continued till identification of the circular fibers of the bladder neck. The mucosa of the bladder neck was then cut open. The 2 lateral lobes of the prostate were also enucleated in the similar manner from the apex to the bladder neck by separating them from the capsule in clockwise or counter-clockwise fashion. Simultaneous hemostasis should be sustained to maintain clear view. After having enucleated most of the prostate adenoma from the surgical capsule, we started to respect the enucleated adenoma from the top with little adenoma still attached to the bladder neck. At that moment, most of the blood supply had been coagulated. The adenoma could be rapidly and completely resected with a resection loop without much bleeding.

When the resection was complete, all the prostate chips were washed out with Ellik and sent for histology. Twenty two French Foley catheter was inserted. Bladder irrigation continued till urine clear, adequate analgesic was prescribed to prevent bladder spasm.^[5,7]^

The weights of the prostate chips, operation times, intraoperative complications such as perforation of the capsule and intraoperative blood transfusion of these 2 groups were recorded and compared.

For the B-TURP group we followed the next steps:

Step 1: After the panendoscopy, B-TURP starts with resection of the bladder neck and proximal part of the transitional zone circumferentially. Special precaution is needed to avoid the damage of ureteric orifices during resection. Concurrent hemostasis is needed to maintain clear view.
Step 2: Resection of distal part of the transitional zone proximal to the Verumontanum circumferentially. Special precaution is needed to avoid damage of the external sphincter anteriorly.
Step 3: Evacuation of the prostate chips by Ellik after creation of patent prostatic channel.
Step4: The 22 or 24 French 3-way Foley insertion after hemostasis is encertained. Bladder irrigation starts after the Foley insertion.

Follow-up: The patients were followed up at 1, 3, and 6 months after the operations. Complications, post void residual urine (PVRU), maximum flow rate (Qmax), International Prostatic Symptoms Scale (IPSS), and quality of life (QOL) were recorded.

Statistical methods: All the data were analyzed using SPSS version 22.0 (SPSS Inc. Chicago, Illinois). Sample *t* test, paired *t* test and Chi-Squared test were used. The statistically significant difference was *P* < .05.

## 3 Result

General clinical data: The ages of the patients ranged from 64 to 92 years, with mean age of 72.31 ± 6.02 years. The course of the disease ranged from 48 to 342 months. The volumes of the prostates ranged from 80 to 125 mL, with mean volume of 98.32 ± 11.50 mL. There were no significant differences of mean age and mean volume between these 2 groups (*P* = .407, *P* = .302) as shown in Table 1.

**Table 1:** Comparison of the general data between patients of BipoLEP group and B-TURP.

Group	Number of cases	Mean age (yr)	Mean volume (mL)
BipoLEP	72	71.35 ± 9.3	99.14 ± 9.5
B-TURP	56	73.26 ± 9.4	95.08 ± 10.93
*t* value		0.796	1.196
*P* value		.407	.302

Perioperative data and complications. There was no significant difference in operative time between these 2 groups (*P* = .065). The duration of continuous bladder irrigation and indwelling catheter and hospitalization days after operation of the BipoLEP group were significantly less than those in B-TURP group (*P* = .044, *P* = .0320, *P* = .031, respectively). BipoLEP group had less complications than B-TURP group (*P* = .021) as shown in Table 2. The commonest intraoperative complication was capsular perforation which found in 2 cases BipoLEP and 3 cases in B-TURP. Any of these capsular perforations was recognized by massive bleeding managed by catheter balloon traction. Except for 1 patient in B-TURP group who needed blood transfusion all perforations were not distressed and no further intervention was required. Urethral stricture was reported after 1 month of surgery in B-TURP group. Patients stated urinary retention, cystoscopy had shown contracture of the bladder neck, so the patient underwent urethral dilation which accompanied by urethral catheterization, and 2 weeks after that the catheter was removed without any more managements, no TUR syndrome and perforation of the bladder was detected. After catheter withdrawal, more patients in B-TURP group experienced transient incontinence, but most improved spontaneously within 3 months

**Table 2:** Comparison of perioperative data and complications between patients of BipoLEP group and B-TURP.

Group	Operation time (min)	Resection quality (g)	Resection ratio (%)	Irrigation time (h)	Indwelling time (d)	Hospitalization time (d)	Complications [case (%)]
BipoLEP	93.7 ± 27.5	64.2 ± 22.1	64.17 ± 10.15	20.7 ± 6.5	4.3 ± 1.5	5.2 ± 1.4	3 (4.17)
B-TURP	89.8 ± 22.4	52.7 ± 28.6	54.45 ± 9.62	29.6 ± 8.3	5.6 ± 2.1	6.5 ± 1.9	9 (16.07)
T*x*^2^ value	1.805	2.455	2.395	2.075	2.193	2.206	5.254^∗^
*P* value	.065	.018	.025	.044	.032	.031	.021

There were no significant differences of preoperative and 3-month postoperative parameters, including: PVRU, Qmax, IPSS, and QOL between these 2 groups (*P* > .05) as shown in Table 3. One case in BipoLEP group had stress urinary incontinence but recovered 20 days after the pelvic floor exercise, 3 cases in B-TURP group had stress urinary incontinence but recovered after the pelvic floor exercise. In B-TURP group, 2 cases needed to be retreated, 1 case required minimally invasive surgery while another case required medication.

**Table 3:** Follow up of 3 months after operation.

	PVRU (mL)		Qmax (mL/s)		I-PSS (points)		QOL (points)	
Group	Preoperative	Postoperative	Preoperative	Postoperative	Preoperative	Postoperative	Preoperative	Postoperative
BipoLEP	160.5 ± 32.3	10.5 ± 2.4	7.1 ± 3.2	22. 6 ± 5.1	23.9 ± 5.0	5.9 ± 3.3	5.1 ± 0.7	1.2 ± 0.6
B-TURP	155.7 ± 44.5	12.2 ± 3.1^∗^	6.5 ± 2.9	20.3 ± 4.5^∗^	25.1 ± 4.4	6.2 ± 4.1^∗^	4.8 ± 0.8	1.3 ± 0.5^∗^
Tx^2^value	0.771	0.855	1.332	1.128	1.155	0.743	1.431	1.514
*P* value	.402	.393	.186	.253	.314	.436	.165	.149

## 4 Discussion

BPH is a common disease in middle-aged and elderly male patients. The incidence of BPH increases after 40 years of age. The prevalence of BPH over 85 years old can reach up to 90%.^[8]^ Traditionally, surgical treatment of BPH is TURP. However, it is associated with more bleeding, higher chance of TUR syndrome and residual prostate adenoma as compared to BipoLEP, particularly for large prostate larger than 80 g.^[9]^ Studies also questioned the status of its “gold standard” in the surgical treatment of the BPH.^[10]^ The third generation of prostate electrosurgical equipment, bipolar plasma electrosurgical apparatus, uses isotonic solution as irrigation agent effectively minimizes the occurrence of TUR syndrome. Effective hemostatic effect improves the surgical outcome of the surgery. Studies also showed that the bipolar system caused less damage to the peripheral tissues including extracapsular neovascular bundle, causing less postoperative erectile dysfunction. So, it is now widely used in minimally invasive surgery of the prostate.^[11]^ However, studies showed B-TURP had more residual prostate adenoma and therefore higher retreatment rate as compared to BipoLEP, particularly in large prostate larger than 80 g. So, prostate size is a very important parameter for choosing the treatment of choice for BPH.^[12]^ In our study, we also showed more prostate tissue resected in the BipoLEP group

Zhang et al did a meta-analysis of comparison between TURP and BipoLEP, which included 26 randomized control trials involving 3283 patients. Similar to the results of our study, there were no significant differences of IPSS, Qmax, and QOL at 1, 3, and 6 months after the operations.^[10]^

Hirasawa et al also had a retrospective review on 110 patients which showed shorter catheterization time and hospital stay in BipoLEP group as compared to B-TURP group.^[13]^ Liu and his colleagues performed 1100 cases of BipoLEP with prostates sizes ranged from 35 to 256 g.^[7]^ The mean operation time was 61.5 minutes (enucleation time: 15.5 minutes, resection time: 46 minutes). Only 0.8% cases required blood transfusion. Mean catheter time and hospital stay were 1.8+/− 0.4 days and 5.3+/−2.3 days, respectively. The mean follow-up time was 4.3 years. There was a significant improvement in Qmax, PVRU, IPSS, and QOL scores with few complications. In view of these findings, Liu and his colleagues suggested the indication of BipoLEP could be expanded for prostate glands of any sizes. Rao and his partners retrospectively investigated on the perioperative data of 326 patients underwent BipoLEP according to the prostate sizes.^[12]^ They found that the prostate size had no effect on the perioperative outcome and 12-month postoperative voiding outcomes.

The key to shorten the operation time of BipoLEP lies on the accuracy of finding the surgical plane between the prostatic adenoma and the capsule. Simultaneous hemostasis is also very important to make the view as clear as possible during the whole procedure. Early apical dissection and avoidance of forceful blunt dissection are very importance to prevent damage of the external urethral sphincter.^[14]^

In our BipoLEP group, we enucleated the prostate adenoma in 3 lobes technique. We started with enucleation of the median lobe from 5 to 7 o’clock. Then, we enucleated the left lobe in anticlockwise manner and finally the right lobe in clockwise manner. For beginners, this method can be used as a proficient process of improving enucleation technique, laying a good foundation which results in shortening the learning curve of the BipoLEP.^[10]^

As valuable options for the management of BPH, holmium laser prostate enucleation (HoLEP), and current-based treatment (BipoLEP) are equally valued. This was mainly based on the results of 2 meta-analyses in randomized controlled trials comparing open prostatectomy with either HoLEP or BipoLEP.^[15,16]^ Similarly, comparison of HoLEP vs BipoLEP in Guo et al review did not show the superiority of 1 method over the other.^[17]^ However, the advantages of the BipoLEP over other methods include:

1. Shorter learning curve.
2. All Urologists have familiarized with the setup, instrument of the BipoLEP as used in the conventional TURP.
3. Urologists can change back to the TURP directly if failed BipoLEP of any reasons.
4. Lower cost as the resection loop of the BipoLEP is cheaper and can be reusable.
5. No special electricity plug supply is needed as compared to the laser enucleation.

There was several limitations of this study. Firstly, it failed to evaluate the effect of surgery on the serum sodium and other electrolytes without statistics of absorption of irrigation fluid during the operation. Secondly, there was no data to compare the change of prostate specific antigen (PSA) before and after the surgery in both groups. In future studies, the case number in each group should be increased and the above-mentioned deficiencies should be addressed.

In conclusion, this study showed that BipoLEP technique was more advantageous due to the more desirable profile, described by statistically meaningful variations in period of continuous bladder irrigation and indwelling catheter and hospitalization days. The enucleation group has had a reduced rate of adverse effects and early complications, also it was conducive to the earlier recovery of the patients after BipoLEP.

## Author contributions

**Conceptualization:** Mohammed Abdulwahab Al-radhi.

**Data curation:** Mohammed Abdulwahab Al-radhi.

**Formal analysis:** Mohammed Abdulwahab Al-radhi.

**Methodology:** Mohammed Safi, Khaled M Al-Kohlany, Amr Al-Najar.

**Project administration:** Mohammed Abdulwahab Al-radhi, Mohammed Safi.

**Software:** Mohammed Abdulwahab Al-radhi, Hesham Al-sharani.

**Supervision:** Khaled M Al-Kohlany, XianCheng Li, Chao Wang.

**Visualization:** Abdullah Al-danakh.

**Writing – original draft:** Mohammed Abdulwahab Al-radhi.

**Writing – review & editing:** Mohammed Abdulwahab Al-radhi, Lo Ka Lun.
